# Effectiveness of Sodium Bicarbonate Infusion on Mortality in Critically Ill Children With Metabolic Acidosis

**DOI:** 10.3389/fphar.2022.759247

**Published:** 2022-03-17

**Authors:** Huabin Wang, Rui Liang, Tianqi Liang, Songyao Chen, Yulong Zhang, Lidan Zhang, Chun Chen

**Affiliations:** ^1^ Division of Hematology/Oncology, Department of Pediatrics, The Seventh Affiliated Hospital of Sun Yat-Sen University, Shenzhen, China; ^2^ Department of Pediatric Intensive Care Unit, The Seventh Affiliated Hospital of Sun Yat-Sen University, Shenzhen, China; ^3^ Department of Colorectal Surgery, The Third Affiliated Hospital of Kunming Medical University, Kunming, China; ^4^ Center of Digestive Disease, The Seventh Affiliated Hospital of Sun Yat-Sen University, Shenzhen, China; ^5^ Scientific Research Center, The Seventh Affiliated Hospital of Sun Yat-Sen University, Shenzhen, China

**Keywords:** sodium bicarbonate, pediatric intensive care unit, metabolic acidosis, prognostic value, cohort study

## Abstract

**Objective:** Metabolic acidosis often occurs in the paediatric intensive care unit (PICU). Although sodium bicarbonate (SB) has been widely used in paediatrics, data on the effect of SB on children with metabolic acidosis in the PICU are scarce.

**Methods:** Patients with metabolic acidosis who were treated with SB within 48 h of PICU admission were screened. Multivariate logistic regression, subgroup analysis, and propensity score matching (PSM) were used to investigate the relationships between SB infusion and clinical outcomes.

**Results:** A total of 1,595 patients with metabolic acidosis were enrolled in this study. In the multivariate logistic regression model, SB infusion was not correlated with in-hospital mortality (odds ratio (OR) 0.87, 95% confidence interval (CI) 0.47–1.63, *p* = 0.668), but was significantly correlated with hypernatraemia (OR 1.98, 95% CI 1.14–3.46, *p* = 0.016), hypokalaemia (OR 2.01, 95% CI 1.36–2.96, *p* < 0.001), and hypocalcaemia (OR 4.29, 95% CI 2.92–6.31, *p* < 0.001). In the pH value, lactate level, acute kidney injury level, age grouping, and anion gap level subgroups, the ORs for SB and in-hospital mortality were not statistically significant. After PSM, the results remained unchanged.

**Conclusion:** SB infusion does not reduce the in-hospital mortality of severely ill children with metabolic acidosis and increases the risk of hypernatraemia, hypokalaemia, and hypocalcaemia. More effort should be focused on eliminating the causes of metabolic acidosis rather than SB infusion.

## Introduction

Metabolic acidosis is a disorder of the body’s acid-base balance characterized by a primary decrease in plasma bicarbonate concentration (BC) caused by an increase in hydrogen ions or a loss of bicarbonate in the extracellular fluid (Fujii et al., 2019). Metabolic acidosis can be divided into lactic acidosis, ketoacidosis, hyperchloremic acidosis and so on and has a high incidence in the paediatric intensive care unit (PICU) (Kraut, 2018). Diseases such as sepsis, severe hypoxemia, and cardiogenic shock are the main causes of metabolic acidosis (Velissaris et al., 2015; Haas et al., 2016). Metabolic acidosis can cause haemodynamic instability, decrease myocardial contractility and arterial vasodilation, decrease the cellular oxygen supply and mitochondrial oxygen consumption, impair responsiveness to catecholamines, and induce insulin resistance, leading to increased mortality (Schotola et al., 2012; Kraut and Madias, 2014).

At present, the most basic treatment for metabolic acidosis is to eliminate the underlying causes, such as treating infections and increasing the oxygen supply. However, some institutions have used sodium bicarbonate (SB) or other alkaline drugs after ineffective treatments. Theoretically, SB infusion can neutralize excess acid, thereby restoring the blood pH (Loomba et al., 2020). However, several studies have shown that SB infusions in critically ill patients with metabolic acidosis do not reduce mortality and increases the risk of adverse reactions. It is highly controversial whether SB should be infused in critically ill patients with metabolic acidosis. Existing studies mostly focus on adults, and there have been few reports on severely ill children; therefore, PICU physicians have no clear guidance regarding the use of SB. As such, the purpose of this study was to evaluate whether SB infusion could improve the prognosis of critically ill children with metabolic acidosis.

## Materials and Methods

### Database Introduction

The Paediatric Intensive Care (PIC) database is a completely open, single-centre, bilingual Chinese-English database developed by the Children’s Hospital of Zhejiang University School of Medicine (Zeng et al., 2020). It is a database specifically for PICU patients and includes diagnostic, testing, and monitoring data for non-adult patients aged 0–18 years from multiple intensive care units (ICUs). This database contains information regarding 13,499 hospital stays for 12,881 paediatric patients. The PIC database is based on the widely used Medical Information Mart for Intensive Care (MIMIC) database and can be downloaded and used after registration, application, and certification. The data used in this study were collected in adherence to Health Insurance Portability and Accountability Act (HIPAA) standards. This project was approved by the Institutional Review Committee of the Children’s Hospital of Zhejiang University School of Medicine and exempt from obtaining patient informed consent.

### Inclusion and Exclusion Criteria

The inclusion criteria were as follows: 1) patients between 1 month and 18 years old; 2) if a patient was hospitalized multiple times, only information from the first hospitalization was selected; and 3) patients with metabolic acidosis in the first 48 h of ICU admission (pH < 7.35, BC < 22 mmol/L).

The exclusion criteria were as follows: 1) patients in the neonatal intensive care unit; 2) lack of chart event data in the database; 3) patients undergoing cardiac surgery; and 4) patients with combined respiratory acidosis (partial pressure of carbon dioxide (PaCO_2_) > 50 mmHg).

If pH, BC, and PaCO_2_ were measured multiple times within the first 48 h of ICU entry, the minimum pH and BC values and the maximum PaCO_2_ value during this period were used.

### Data Extraction and Definition of the Primary and Secondary Outcomes

The extracted demographic information included age, sex, and type of ICU admission; experimental variables included white blood cell (WBC) count, platelet (PLT) count, activated partial thromboplastin time (APTT), anion gap, and lactic acid concentration, and the initial values at ICU admission were used for the above variables. Comorbidities included anaemia, hypertension, acute kidney injury (AKI), liver dysfunction, and ketoacidosis. All comorbidities were diagnosed within 48 h of ICU admission. We used the pROCK criterion, which defines AKI as an increase in creatinine levels of ≥20 μmol/L and ≥30% within 7 days (Xu et al., 2018). The pROCK classifies AKI stages 2 and 3 as creatinine increases of ≥40 μmol/L and ≥60% and ≥80 μmol/L and ≥120%, respectively. The diagnostic criteria of other comorbidities are provided in Supplementary Table S1. The percentage of missing values of each variable was less than 5%; therefore, the mean or median was used to replace the missing value.

The primary outcome measure was in-hospital mortality, defined as death during hospitalization. The secondary outcome measures included hypernatraemia, hypokalaemia, hypocalcaemia, hospitalization length of stay (LOS) and 30-days mortality. Only hypernatraemia (serum sodium >150 mmol/L), hypokalaemia (serum potassium <3.5 mmol/L), and hypocalcaemia (free calcium <1.2 mmol/L) that occurred after the use of SB were counted. The maximum sodium value and minimum potassium and calcium values were selected.

### Statistical Analysis

Continuous variables with a normal distribution are presented as the mean ± standard deviation, and continuous variables with a nonnormal distribution are presented as the median (quartile). Student’s t test, the Wilcoxon rank sum test, or the chi-squared test was used to compare differences between two groups. A backward stepwise method was used to screen the covariates for multivariate logistic regression. Considering that pH, lactate, and AKI are important factors that affect the use of SB in clinical practice and that different physiopathological situations may arise at different ages and anion gaps, the above factors were used for subgroup analysis. To verify the interaction between SB and these variables, multiplicative interaction terms were incorporated in the regression model.

Propensity score matching (PSM) was used to minimize the influence of confounding factors that may cause bias in the results. PSM scores were assigned based on the probability of patients receiving SB treatment and were estimated using a multivariate logistic regression model. The 1:1 nearest neighbour matching algorithm was used for matching, and the caliper value was set to 0.05. Based on Table 1, the following variables were selected to generate the PSM score: age, ICU type, APTT, anion gap, anaemia, hypertension, and AKI.

**TABLE 1:** Comparisons of the baseline characteristics between patients with or without sodium bicarbonate use.

Variable	All patients	Non-SB group	SB group	*P*
	(n = 1,595)	(n = 1,045)	(n = 550)	
Age, months	17 (5–50)	17 (5–52)	16 (5–43)	0.593
Age, n (%)	—	—	—	0.077
<12 months	692 (43.4)	452 (43.3)	240 (43.6)	—
≥12 months and <60 months	598 (35.6)	365 (34.9)	203 (36.9)	—
≥60 months and <120 months	207 (13.0)	131 (12.5)	76 (13.8)	—
≥120 months	128 (8.0)	97 (9.3)	31 (5.6)	—
Male, n (%)	904 (56.7)	598 (57.2)	306 (55.6)	0.543
ICU type, n (%)	—	—	—	0.033
PICU	417 (26.1)	291 (27.8)	126 (22.9)	—
SICU	1,178 (73.9)	754 (72.2)	424 (77.1)	—
**Laboratory data**	—	—	—	—
WBC (<4 or >12, 10^9^/L)	679 (42.6)	435 (41.6)	244 (44.4)	0.293
Platelet (<100, 10^9^/L)	100 (6.3)	66 (6.3)	34 (6.2)	0.916
Lactate (≥2.0, mmol/L)	625 (39.2)	412 (39.4)	213 (38.7)	0.786
APTT (>45, s)	246 (15.4)	186 (17.8)	60 (10.9)	<0.001
PH (<7.2)	81 (5.1)	56 (5.4)	25 (4.5)	0.482
BC (<14, mmol/L)	173 (10.8)	113 (10.8)	60 (10.9)	0.953
Anion gap (8–16, mmol/L)	510 (32.0)	404 (38.7)	106 (19.3)	<0.001
**Comorbidities, n (%)**				
Anemia	1,093 (68.5)	693 (66.3)	400 (72.7)	0.009
Hypertension	349 (21.9)	183 (17.5)	166 (30.2)	<0.001
AKI	181 (11.3)	133 (12.7)	48 (8.7)	0.017
AKI stage ≥2	48 (3.0)	16 (1.5)	32 (5.8)	0.865
Liver dysfunction	309 (19.4)	198 (18.9)	111 (20.2)	0.553
Diabetic ketoacidosis	117 (7.3)	76 (7.3)	41 (7.5)	0.895
**Clinical outcome**				
Hypernatremia	59 (3.7)	31 (3.0)	28 (5.1)	0.033
Hypokalaemia	121 (7.6)	64 (6.1)	57 (10.4)	0.002
Hypocalcemia	135 (8.5)	51 (4.9)	84 (15.3)	<0.001
Hospital LOS (day)	11 (7–18)	11 (7–18)	12 (6–19)	0.667
30 days mortality, n (%)	58 (3.6)	40 (3.8)	18 (3.3)	0.574
Hospital mortality, n (%)	61 (3.8)	44 (4.2)	17 (3.1)	0.268

AKI, acute kidney injury; APTT, activated partial thromboplastin time; BC, bicarbonate concentration; LOS, length of stay; PICU, pediatric intensive care unit; SB, sodium bicarbonate; SICU, surgery intensive care unit; WBC, white blood cell.

A *p* value <0.05 was considered statistically significant. Statistical analysis was performed using STATA (V.16), SPSS (V.24), and R (V.3.6.3).

## Results

### Baseline Characteristics

**FIGURE 1:**
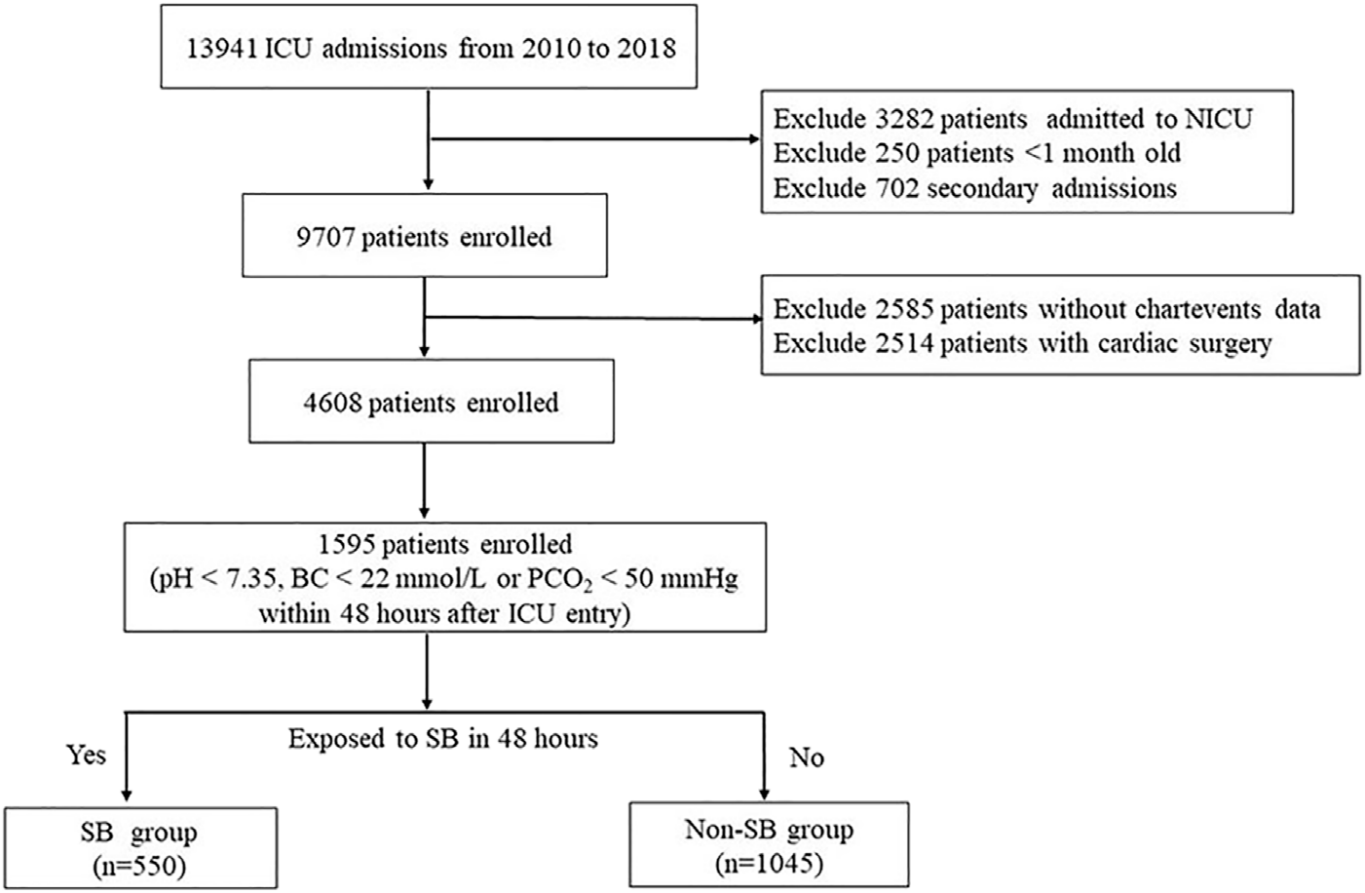
Flow diagram of patient recruitment. NICU, neonatal intensive care unit; SB, sodium bicarbonate.

A total of 1,595 patients were enrolled; the study flowchart is shown in Figure 1. Table 1 provides the baseline characteristics data. The overall median age was 17 months. A total of 56.7% of patients were male, and the in-hospital mortality was 3.8%. Comparisons between the SB and non-SB groups indicated that there were significant differences in ICU type, APTT, anion gap, anaemia, hypertension, and AKI. The occurrence of hypernatraemia (5.1 vs. 3.0%, *p* = 0.033), hypokalaemia (10.4 vs. 6.1%, *p* = 0.002), and hypocalcaemia (15.3 vs. 4.9%, *p* < 0.001) in the SB group was higher than that in the non-SB group. However, there were no significant differences between the two groups in LOS, 30-days mortality, or in-hospital mortality.

### Primary Outcome

**FIGURE 2:**
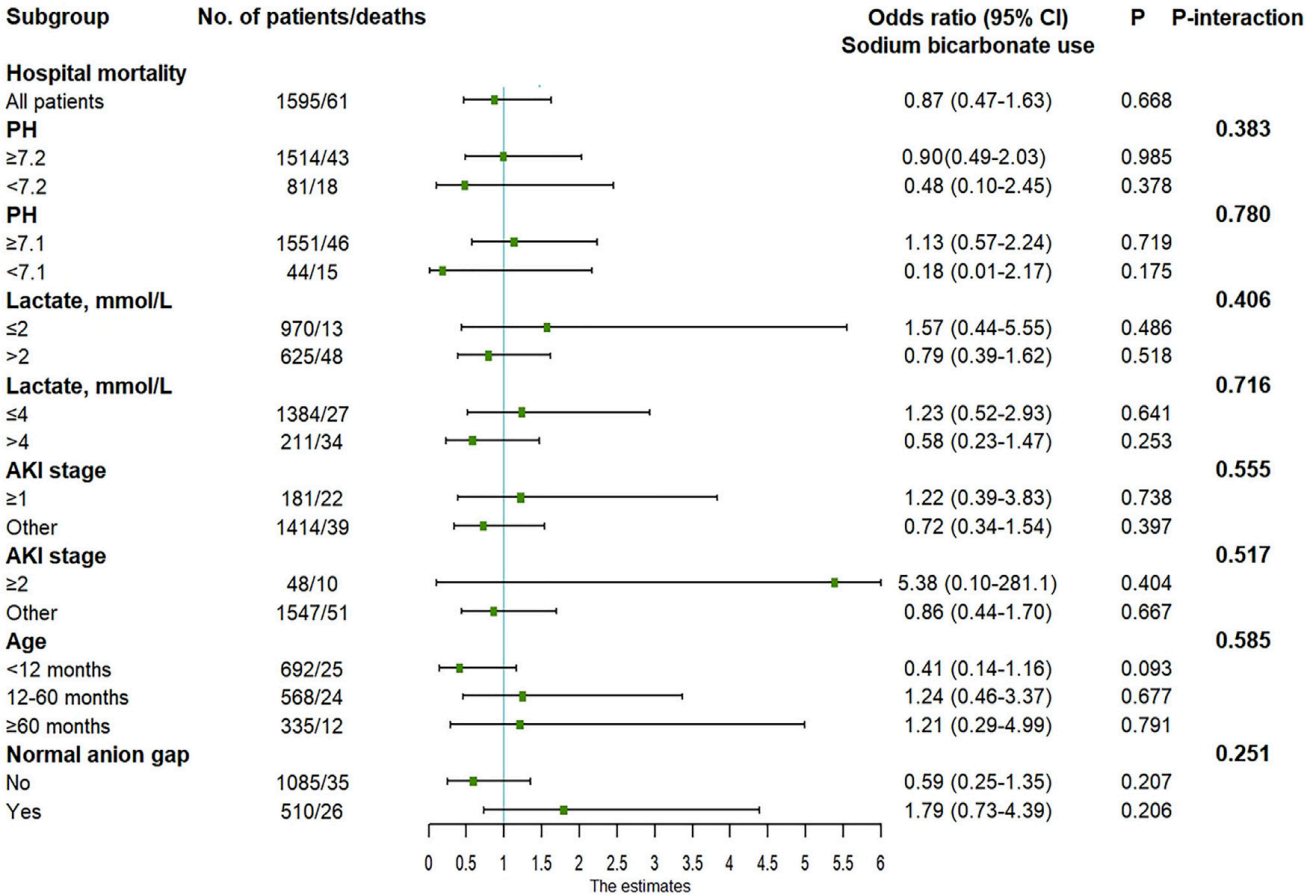
Adjusted odds ratio in the subgroup analysis. AKI, acute kidney injury; CI, confidence interval.

The results of univariate and multivariate logistic regressions with in-hospital mortality as the outcome variable are shown in Table 2. Regardless of univariate analysis or multivariate analysis, there was no significant correlation between SB use and in-hospital mortality. In the multivariate analysis, ICU type, lactate level, APTT, and AKI were independent risk factors for in-hospital death. Subgroup analysis was performed based on pH, lactate level, AKI, age and anion gap (Figure 2). When constructing a logistic regression model for the subgroup with age ≥120 months, the small number of deaths in this subgroup precluded statistical analysis. Therefore, we set age ≥60 months as a subgroup. The odds ratios (ORs) for SB and in-hospital death in the different subgroups were not statistically significant. In addition, the *p-interaction* between SB and pH, lactate, AKI, age, or anion gap was greater than 0.05.

**TABLE 2:** Logistic regressions of sodium bicarbonate use for in-hospital mortality.

	Crude OR	95% CI	*P*	Adjusted OR	95% CI	P
Age						
<12 months	Ref.-			Ref		
≥12 months and <60 months	1.18	0.67–2.08	0.576	1.26	0.67–2.37	0.472
≥60 months and <120 months	1.07	0.48–2.42	0.866	1.12	0.46–2.69	0.805
≥120 months	0.86	0.29–2.52	0.784	0.58	0.18–1.83	0.351
Gender (female)	0.84	0.50–1.42	0.523	0.67	0.39–1.17	0.160
ICU type (PICU)	6.31	3.65–10.91	<0.001	3.54	1.96–6.42	<0.001
WBC (<4 or >12, 10^9^/L)	2.00	1.19–3.36	0.009	1.54	0.88–2.67	0.127
Platelet (<100, 10^9^/L)	3.57	1.80–7.10	<0.001	1.45	0.66–3.18	0.358
Lactate (≥2.0, mmol/L)	6.12	3.29–11.40	<0.001	4.39	2.32–8.31	<0.001
APTT (>45, s)	3.83	2.25–6.53	<0.001	2.30	1.30–4.06	0.004
Anion gap (8–16, mmol/L)	1.61	0.96–2.71	0.071	1.04	0.58–1.84	0.903
Anemia	0.94	0.54–1.62	0.822	0.87	0.48–1.57	0.635
Hypertension	0.45	0.20–1.00	0.051	0.54	0.23–1.23	0.142
Acute kidney injury	4.88	2.82–8.44	<0.001	2.26	1.24–4.11	0.007
Liver dysfunction	3.53	2.09–5.94	<0.001	1.50	0.83–2.70	0.180
Diabetic ketoacidosis	2.29	1.10–4.76	0.027	1.19	0.53–2.66	0.673
Sodium bicarbonate use	0.73	0.41–1.28	0.270	0.87	0.47–1.63	0.668

APTT, activated partial thromboplastin time; CI, confidence interval; OR, odds ratio; PICU, pediatric intensive care unit; WBC, white blood cell.

### Secondary Outcomes

Multivariate logistic regression was performed using hypernatraemia, hypokalaemia, hypocalcaemia, LOS, and 30-day mortality as the outcome variables, as shown in Table 3. LOS was converted to a dichotomous variable based on the median value (11.7 days). The use of SB was associated with an increased risk for hypernatraemia (OR 1.98, 95% CI 1.14–3.46, *p* = 0.016), hypokalaemia (OR 2.01, 95% CI 1.36–2.96, *p* < 0.001) and hypocalcaemia (OR 4.29, 95% CI 2.92–6.31, *p* < 0.001). The OR values for other covariates are provided in Supplementary Table S2.

**TABLE 3:** Multivariate logistic regression of sodium bicarbonate infusion for secondary outcome.

Secondary outcome	OR	95% CI	*P*
Hypernatremia	1.98	1.14–3.46	0.016
Hypokalaemia	2.01	1.36–2.96	<0.001
Hypocalcemia	4.29	2.92–6.31	<0.001
Longer hospital LOS (>11.3 days)	0.96	0.77–1.20	0.726
30 days mortality	1.03	0.55–1.90	0.937

CI, confidence interval; LOS, length of stay; OR, odds ratio.

### PSM Results

Utilizing the 1:1 matching algorithm, there were 518 matched pairs (Table 4). The overall quality of the matched samples was assessed by examining the propensity scores between groups (Supplementary Figure S1). All covariates were balanced between the two groups. There were no significant differences in in-hospital mortality, 30-days mortality, or LOS between the two groups; however, in the SB group, there were increased incidences of hypernatraemia (5.4 vs. 2.7%, *p* = 0.027), hypokalaemia (10.6 vs. 6.0%, *p* = 0.007), and hypocalcaemia (15.8 vs. 6.2%, *p* < 0.001).

**TABLE 4:** Comparisons of the covariates after propensity score matching.

Variable	All patients	Non-SB group	SB group	*P*
	(n = 1,036)	(n = 518)	(n = 518)	
Age, months	17 (5–44)	17 (5–43)	17 (5–47)	0.525
ICU type, n (%)	—	—	—	1.000
PICU	240 (23.2)	120 (23.2)	120 (23.2)	—
SICU	796 (76.8)	398 (76.8)	398 (76.8)	—
APTT (> 45, s)	115 (11.1)	55 (10.6)	60 (11.6)	0.621
Anion gap (8–16, mmol/L)	207 (20.0)	101 (19.5)	106 (20.5)	0.698
Comorbidities, n (%)				
Anemia	735 (71.0)	367 (70.9)	368 (71.0)	0.945
Hypertension	261 (25.2)	127 (24.5)	134 (25.9)	0.616
Acute kidney injury	92 (8.9)	44 (8.5)	48 (9.3)	0.662
Clinical outcome				
Hypernatremia	42 (4.1)	14 (2.7)	28 (5.4)	0.027
Hypokalaemia	86 (8.3)	31 (6.0)	55 (10.6)	0.007
Hypocalcemia	114 (11.0)	32 (6.2)	82 (15.8)	<0.001
Hospital LOS (day)	12 (7–19)	12 (7–18)	12 (6–19)	0.767
30 days mortality, n (%)	39 (3.8)	22 (4.3)	17 (3.3)	0.414
Hospital mortality, n (%)	40 (3.9)	24 (4.6)	16 (3.1)	0.197

APTT, activated partial thromboplastin time; LOS, length of stay; PICU, pediatric intensive care unit; SB, sodium bicarbonate; SICU, surgery intensive care unit.

### Sensitivity Analysis

To test the reliability of the results after PSM, different covariates were included for the sensitivity analysis. As seen in Table 2, the relationships between the use of SB and clinical outcomes were confounded to some extent by some experimental variables, such as WBC count, PLT count, and lactate level. Therefore, these three variables were added for PSM; there were no significant differences in the covariates after PSM (Supplementary Table S3, Supplementary Figure S2), and the results remained stable.

## Discussion

The main purpose of this study was to evaluate the effect of SB infusion on the prognosis of PICU patients with metabolic acidosis. To the best of our knowledge, this is the largest study on this subject ever conducted in a PICU. The results indicated that SB infusion was not correlated with changes in mortality in critically ill children with metabolic acidosis, even in subgroups with pH < 7.1, AKI and hyperlactacidaemia. Additionally, SB infusion was associated with an increased risk of hypernatraemia, hypokalaemia, and hypocalcaemia.

Currently, whether SB infusion improves survival in patients with metabolic acidosis is debated. Several studies have shown that SB treatment does not improve patient prognosis but increases the incidence of adverse reactions and even mortality. Loomb et al. conducted a systematic review and meta-analysis, which included 341 children from six studies, to determine whether SB treatment can improve haemodynamics, gas exchange, and blood oxygen saturation in infants with metabolic acidosis (Loomba et al., 2020). The results suggested that the use of SB did not improve oxygen saturation as measured by pulse oximetry, heart rate, blood pressure (BP), pH, or oxygen partial pressure. Kim et al. retrospectively analysed 103 patients with lactic acidosis and found that the use rate of SB in the deceased group was higher than that in the survival group (*p* = 0.006); multivariate logistic regression suggested that the administration of SB was an independent risk factor for in-hospital death (OR 6.27, 95% CI 1.1–35.78) (Kim et al., 2013). In terms of basic research, there are two experiments with dogs with lactic acidosis. Compared with those in dogs in the control group, the blood pH and mortality did not improve in dogs that received SB treatment; however, BP and cardiac output decreased (Arieff et al., 1982; Graf et al., 1985).

It seems intuitive to add an alkaline agent to acidic blood to increase the pH; however, the actual treatment approach is much more complicated (Quade et al., 2021). The inability of SB to improve the prognosis of children with metabolic acidosis can be explained as follows. First, although SB infusion can increase blood pH in a short period of time (Mintzer et al., 2015), it can also increase CO_2_ production and aggravate intracellular acidosis in children with severe circulatory failure (Kim et al., 2013; Collins and Sahni, 2017). Second, SB treatment can lead to fluid overload and electrolyte disorders, including hypocalcaemia, which in turn affects the function of the heart and vascular smooth muscle (Kimmoun et al., 2015; Drumheller and Sabolick, 2021), resulting in cerebral and cardiovascular haemodynamic fluctuations and functional abnormalities (Aschner and Poland, 2008; Berg et al., 2010; Katheria et al., 2017). Studies have reported that SB infusion can increase the risk of cerebral oedema in paediatric patients with diabetic ketoacidosis (Glaser et al., 2001). Third, SB infusion may change the oxyhaemoglobin saturation relationship and increase the production of lactic acid in anaerobic glycolysis (Forsythe and Schmidt, 2000). The results of this study are consistent with the above view that SB cannot reduce the mortality of children with metabolic acidosis and that SB treatment causes hypernatraemia, hypokalaemia, and hypocalcaemia.

As described above, many studies have shown that patients with metabolic acidosis do not benefit from SB infusion. However, some patients with specific diseases or severe conditions may benefit from SB infusion. In a multicentre, phase III randomized controlled trial (RCT) that included 389 patients with severe metabolic acidaemia (pH ≤ 7.20), there was no significant difference in the survival rate at day 28 between the control group and the SB group. However, for patients with an AKI network score of two to three points, the survival rate in the SB group was higher than that in the control group on day 28 (63 vs. 46%, *p* = 0.028) (Jaber et al., 2018). Zhang et al. retrospectively analysed 1718 sepsis patients with metabolic acidosis in the MIMIC database (500 patients in the SB group and 1,218 patients in the non-SB group) (Zhang et al., 2018). The results indicated that there was no significant difference in mortality between the two groups overall (hazard ratio (HR) 1.04, 95% CI 0.86–1.26, *p* = 0.670) but that SB use benefitted the population with grade 2 or three AKI and pH < 7.2 (HR 0.74, 95% CI 0.51–0.86, *p* = 0.021). However, in this study, for PICU patients, SB infusion did not benefit patients in the severe acidosis (pH < 7.2 or pH < 7.1) and AKI subgroups. Notably, the causes of metabolic acidosis or AKI in children are different from those in adults, and different aetiologies may cause different patient responses to SB treatment (Joannes-Boyau and Forni, 2018).

Our research presents some advantages. Thus far, this is the largest study on the efficacy of SB in the PICU; therefore, enough confounding variables were included, and subgroup analysis was performed. However, this study also has limitations. First, this is a retrospective study. Although PSM and sensitivity scores were used to balance the important confounding factors, there may still be some unknown confounding factors that affected the results. Second, multiple subgroup analyses were conducted, but the small number of cases in the pH < 7.1 subgroup may lead to false negative results. Third, due to limitations with respect to the database, data for some treatments were not available, such as balanced salt solution and haemodialysis, which would affect the acid-base balance of patients. Fourth, because of database limitations, we could not precisely classify the type of metabolic acidosis in each patient. For example, organic acidemias are mostly diagnosed by some sophisticated laboratory tests, such as tandem mass spectrometry and gas chromatography–mass spectrometry, which are not available in databases. Therefore, we could not clarify whether the patient had organic acidemia.

## Conclusion

Acidaemia in critically ill children is a common concern of paediatricians. Based on the conclusions of this study, SB infusion may not be beneficial. More effort should be focused on eliminating the causes of metabolic acidosis rather than SB infusion. Notably, RCTs targeting specific disease populations and different types of acidosis are needed.

## Data Availability

The original contributions presented in the study are included in the article/Supplementary Materials, further inquiries can be directed to the corresponding authors.
