# Supplementary material for: Effectiveness of Sodium Bicarbonate Infusion on Mortality in Critically Ill Children With Metabolic Acidosis
Source: Front Pharmacol. 2022 Mar 17;13:759247. doi: 10.3389/fphar.2022.759247 (PMC8970597; doi:10.3389/fphar.2022.759247)
Supplement: Supplementary file 1 [file DataSheet1.docx]

Supplementary Material

# Supplementary Figures and Tables

## Supplementary Figures


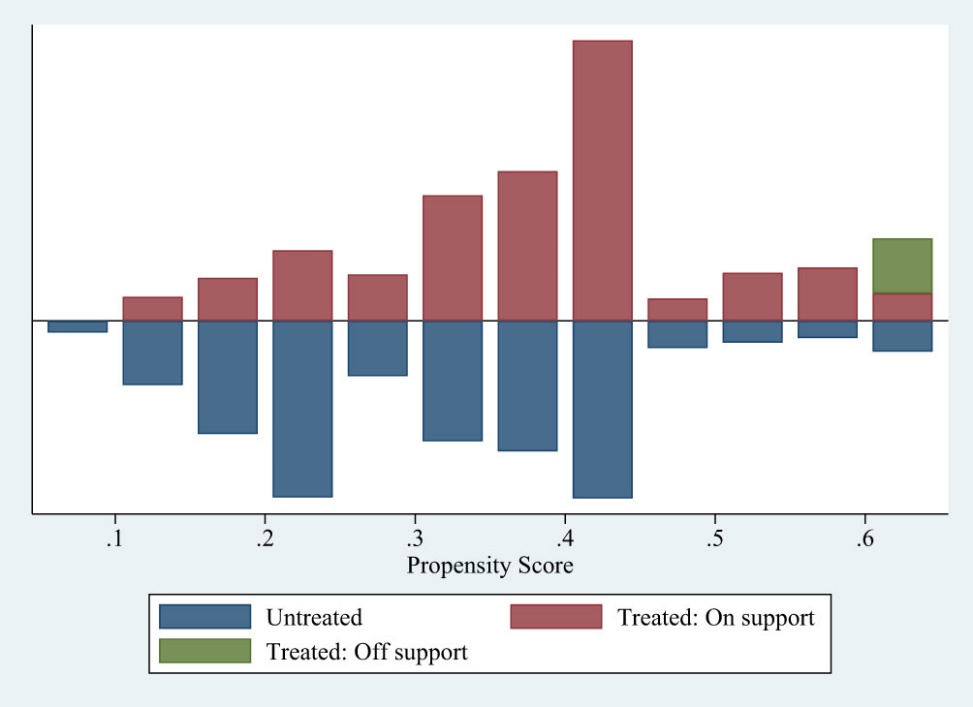


**Supplementary Figure 1.** Propensity scores for the groups of sodium bicarbonate use and non-sodium bicarbonate use before sensitivity analysis.


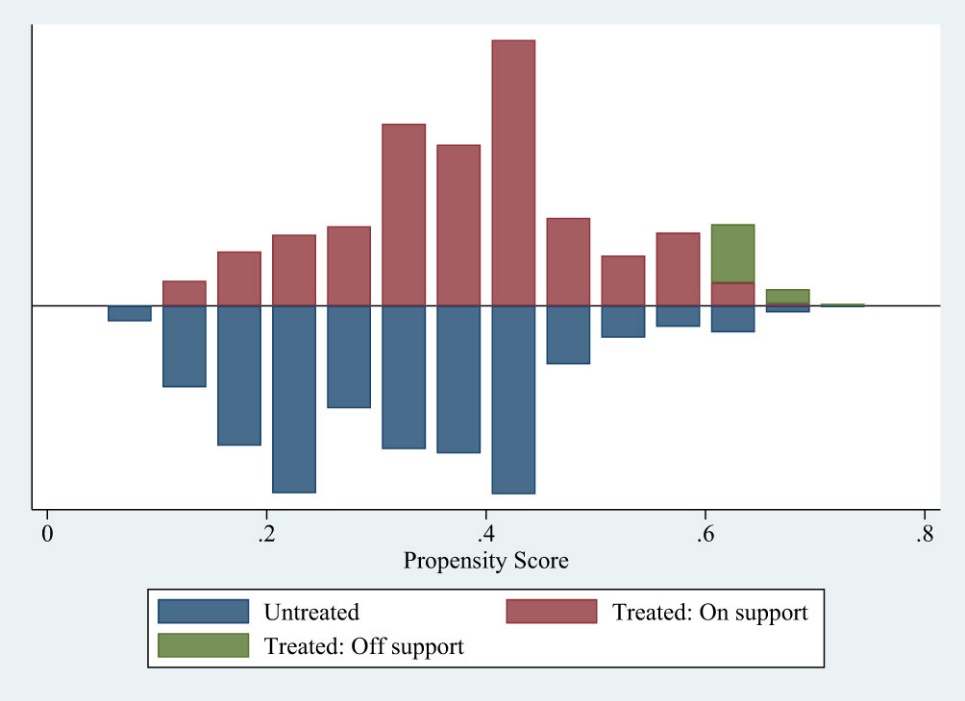


**Supplementary Figure 2.** Propensity scores for the groups of sodium bicarbonate use and non-sodium bicarbonate use after sensitivity analysis.

## 2. Supplementary Tables

**Supplemental Table 1.** Diagnostic criteria for complications.

| **Complications** | **Diagnostic criteria** |
| --- | --- |
| Acute kidney injury | We used the pROCK criterion, which defines acute kidney injury as an increase in creatinine levels of ≥ 20 µmol/L and ≥ 30% within 7 days.  The pROCK classified AKI stages 2 and 3 as SCr increases of ≥ 40 μmol/L and ≥ 60% and ≥ 80 μmol/L and ≥ 120%, respectively. |
| Anemia | 1-4 months < 90 g/L;  4-6 months < 100 g/L;  6-60minths < 110 g/L;  60-144months < 115 g/L;  >144 months < 120 g/L. |
| Hypertension | Systolic pressure > 120 mmHg; diastolic pressure > 80 mmHg. |
| Diabetic ketoacidosis | Blood glucose > 11 mmol/L; venous blood pH < 7.3 or serum bicarbonate < 15 mmol / L; presence of ketone bodies in blood or urine. |
| Liver dysfunction | Total bilirubin > 68.4mmol/L or alanine aminotransferase elevation more than two times the upper value. |

**Supplemental Table 2.1** Logistic regressions of sodium bicarbonate use for hypernatremia.

| **Variable** | **Adjusted OR** | | **95% CI** | | ***P*** | |
| --- | --- | --- | --- | --- | --- | --- |
| Age |  |  | |  | |  |
| < 12 months | Ref. | | | | |  |
| ≥ 12months and < 60 months | 1.63 | 0.83-3.23 | | 0.158 | |  |
| ≥ 60months and < 120 months | 1.72 | 0.72-4.18 | | 0.227 | |  |
| ≥ 120 months | 2.95 | 1.21-7.19 | | 0.018 | |  |
| Gender (female) | 0.92 | 0.52-1.61 | | 0.759 | |  |
| ICU type (PICU) | 4.06 | 2.17-7.60 | | <0.001 | |  |
| WBC (< 4 or > 12, 10^9^/L) | 1.08 | 0.61-1.91 | | 0.797 | |  |
| Platelet (< 100, 10^9^/L) | 0.81 | 0.33-1.97 | | 0.646 | |  |
| Lactate (≥ 2.0, mmol/L ) | 2.03 | 1.13-3.65 | | 0.018 | |  |
| APTT (> 45, s) | 1.72 | 0.89-3.32 | | 0.109 | |  |
| Anion gap (8-16, mmol/L) | 1.29 | 0.72-2.31 | | 0.387 | |  |
| Anemia | 1.98 | 0.99-3.96 | | 0.053 | |  |
| Hypertension | 1.13 | 0.56-2.27 | | 0.737 | |  |
| Acute kidney injury | 3.21 | 1.75-5.88 | | <0.001 | |  |
| Liver dysfunction | 2.14 | 1.20-3.83 | | 0.010 | |  |
| Diabetic ketoacidosis | 1.21 | 0.54-2.68 | | 0.647 | |  |
| Sodium bicarbonate use | 1.98 | 1.14-3.46 | | 0.016 | |  |

APTT, activated partial thromboplastin time; CI, confidence interval; OR, odds ratio; PICU, pediatric intensive care unit; WBC, white blood cell.

**Supplemental Table 2.2**. Logistic regressions of sodium bicarbonate use for hypokalaemia.

| **Variable** | **Adjusted OR** | | **95% CI** | | ***P*** | |
| --- | --- | --- | --- | --- | --- | --- |
| Age |  |  | |  | |  |
| < 12 months | Ref. | | | | |  |
| ≥ 12months and < 60 months | 0.85 | 0.54-1.34 | | 0.483 | |  |
| ≥ 60months and < 120 months | 0.80 | 0.41-1.54 | | 0.502 | |  |
| ≥ 120 months | 1.24 | 0.63-2.47 | | 0.534 | |  |
| Gender (female) | 0.79 | 0.54-1.18 | | 0.249 | |  |
| ICU type (PICU) | 5.11 | 3.45-7.59 | | <0.001 | |  |
| WBC (< 4 or > 12, 10^9^/L) | 1.22 | 0.83-1.80 | | 0.310 | |  |
| Platelet (< 100, 10^9^/L) | 0.85 | 0.42-1.71 | | 0.648 | |  |
| Lactate (≥ 2.0, mmol/L ) | 0.99 | 0.65-1.51 | | 0.949 | |  |
| APTT (> 45, s) | 0.83 | 0.49-1.40 | | 0.487 | |  |
| Anion gap (8-16, mmol/L) | 1.12 | 0.74-1.70 | | 0.600 | |  |
| Anemia | 0.92 | 0.60-1.41 | | 0.702 | |  |
| Hypertension | 0.86 | 0.52-1.40 | | 0.537 | |  |
| Acute kidney injury | 1.18 | 0.71-1.97 | | 0.528 | |  |
| Liver dysfunction | 1.75 | 1.15-2.66 | | 0.009 | |  |
| Diabetic ketoacidosis | 1.01 | 0.51-1.99 | | 0.983 | |  |
| Sodium bicarbonate use | 2.01 | 1.36-2.96 | | <0.001 | |  |

APTT, activated partial thromboplastin time; CI, confidence interval; OR, odds ratio; PICU, pediatric intensive care unit; WBC, white blood cell.

**Supplemental Table 2.3.** Logistic regressions of sodium bicarbonate use for hypocalcemia.

| **Variable** | **Adjusted OR** | | **95% CI** | | ***P*** | |
| --- | --- | --- | --- | --- | --- | --- |
| Age |  |  | |  | |  |
| < 12 months | Ref. | | | | |  |
| ≥ 12months and < 60 months | 1.21 | 0.79-1.86 | | 0.384 | |  |
| ≥ 60months and < 120 months | 0.84 | 0.44-1.61 | | 0.600 | |  |
| ≥ 120 months | 0.78 | 0.36-1.68 | | 0.521 | |  |
| Gender (female) | 0.88 | 0.60-1.29 | | 0.519 | |  |
| ICU type (PICU) | 5.68 | 3.86-8.36 | | <0.001 | |  |
| WBC (< 4 or > 12, 10^9^/L) | 1.30 | 0.89-1.90 | | 0.176 | |  |
| Platelet (< 100, 10^9^/L) | 1.33 | 0.71-2.48 | | 0.379 | |  |
| Lactate (≥ 2.0, mmol/L ) | 0.77 | 0.52-1.16 | | 0.213 | |  |
| APTT (> 45, s) | 0.88 | 0.51-1.52 | | 0.644 | |  |
| Anion gap (8-16, mmol/L) | 1.19 | 0.78-1.82 | | 0.410 | |  |
| Anemia | 0.77 | 0.52-1.15 | | 0.197 | |  |
| Hypertension | 1.13 | 0.71-1.80 | | 0.595 | |  |
| Acute kidney injury | 1.43 | 0.86-2.36 | | 0.169 | |  |
| Liver dysfunction | 1.49 | 0.98-2.27 | | 0.062 | |  |
| Diabetic ketoacidosis | 1.23 | 0.65-2.32 | | 0.526 | |  |
| Sodium bicarbonate use | 4.29 | 2.92-6.31 | | <0.001 | |  |

APTT, activated partial thromboplastin time; CI, confidence interval; OR, odds ratio; PICU, pediatric intensive care unit; WBC, white blood cell.

**Supplemental Table 2.4.** Logistic regressions of sodium bicarbonate use for longer hospital length of stay (> 11.3 days).

| **Variable** | **Adjusted OR** | | **95% CI** | | ***P*** | |
| --- | --- | --- | --- | --- | --- | --- |
| Age |  |  | |  | |  |
| < 12 months | Ref. | | | | |  |
| ≥ 12months and < 60 months | 0.72 | 0.56-0.91 | | 0.007 | |  |
| ≥ 60months and < 120 months | 0.82 | 0.59-1.15 | | 0.255 | |  |
| ≥ 120 months | 0.94 | 0.62-1.43 | | 0.786 | |  |
| Gender (female) | 1.10 | 0.90-1.35 | | 0.361 | |  |
| ICU type (PICU) | 0.41 | 0.32-0.53 | | <0.001 | |  |
| WBC (< 4 or > 12, 10^9^/L) | 1.49 | 1.20-1.84 | | <0.001 | |  |
| Platelet (< 100, 10^9^/L) | 1.02 | 0.65-1.60 | | 0.933 | |  |
| Lactate (≥ 2.0, mmol/L ) | 0.82 | 0.66-1.03 | | 0.091 | |  |
| APTT (> 45, s) | 0.73 | 0.54-0.99 | | 0.045 | |  |
| Anion gap (8-16, mmol/L) | 0.96 | 0.76-1.20 | | 0.688 | |  |
| Anemia | 0.82 | 0.65-1.02 | | 0.080 | |  |
| Hypertension | 1.26 | 0.97-1.62 | | 0.081 | |  |
| Acute kidney injury | 1.25 | 0.88-1.77 | | 0.217 | |  |
| Liver dysfunction | 1.94 | 1.46-2.56 | | <0.001 | |  |
| Diabetic ketoacidosis | 1.66 | 1.11-2.49 | | 0.013 | |  |
| Sodium bicarbonate use | 0.96 | 0.77-1.20 | | 0.726 | |  |

APTT, activated partial thromboplastin time; CI, confidence interval; OR, odds ratio; PICU, pediatric intensive care unit; WBC, white blood cell.

**Supplemental Table 2.5.** Logistic regressions of sodium bicarbonate use for 30 days mortality.

| **Variable** | **Adjusted OR** | | **95% CI** | | ***P*** | |
| --- | --- | --- | --- | --- | --- | --- |
| Age |  |  | |  | |  |
| < 12 months | Ref. | | | | |  |
| ≥ 12months and < 60 months | 1.26 | 0.67-2.35 | | 0.475 | |  |
| ≥ 60months and < 120 months | 0.80 | 0.31-2.11 | | 0.654 | |  |
| ≥ 120 months | 0.60 | 0.19-1.89 | | 0.380 | |  |
| Gender (female) | 0.79 | 0.45-1.38 | | 0.403 | |  |
| ICU type (PICU) | 4.18 | 2.24-7.80 | | <0.001 | |  |
| WBC (< 4 or > 12, 10^9^/L) | 1.48 | 0.84-2.61 | | 0.178 | |  |
| Platelet (< 100, 10^9^/L) | 1.48 | 0.67-3.26 | | 0.336 | |  |
| Lactate (≥ 2.0, mmol/L ) | 3.67 | 1.91-7.04 | | <0.001 | |  |
| APTT (> 45, s) | 1.79 | 0.98-3.29 | | 0.059 | |  |
| Anion gap (8-16, mmol/L) | 0.98 | 0.55-1.77 | | 0.951 | |  |
| Anemia | 0.87 | 0.47-1.64 | | 0.674 | |  |
| Hypertension | 0.59 | 0.25-1.36 | | 0.212 | |  |
| Acute kidney injury | 1.97 | 1.06-3.65 | | 0.032 | |  |
| Liver dysfunction | 1.78 | 0.98-3.24 | | 0.058 | |  |
| Diabetic ketoacidosis | 1.06 | 0.46-2.47 | | 0.893 | |  |
| Sodium bicarbonate use | 1.03 | 0.55-1.90 | | 0.937 | |  |

APTT, activated partial thromboplastin time; CI, confidence interval; OR, odds ratio; PICU, pediatric intensive care unit; WBC, white blood cell.

**Supplemental Table 3.** Comparisons of the covariates after propensity score matching using sensitivity analysis.

| **Variable** | **All patients** | **Non-SB group** | **SB group** | ***P*** |
| --- | --- | --- | --- | --- |
|  | **(n=1028)** | **(n=514)** | **(n=514)** |  |
| **Age, months** | 16 (5-44) | 14 (5-44) | 17 (5-46) | 0.223 |
| **ICU type, n (%)** |  |  |  | 0.883 |
| PICU | 784 (76.3) | 393 (76.5) | 391 (76.1) |  |
| SICU | 244 (23.7) | 121 (23.5) | 123 (23.9) |  |
| **Laboratory data** |  |  |  |  |
| WBC (< 4 or > 12, 10^9^/L) | 445 (43.3) | 218 (42.4) | 227 (44.2) | 0.571 |
| Platelet (< 100, 10^9^/L) | 61 (5.9) | 29 (5.6) | 32 (6.2) | 0.692 |
| Lactate (≥ 2.0, mmol/L) | 391 (38.0) | 196 (38.1) | 195 (37.9) | 0.949 |
| APTT (> 45, s) | 118 (11.5) | 58 (11.3) | 60 (11.7) | 0.845 |
| Anion gap (8-16, mmol/L) | 196 (19.1) | 90 (17.5) | 106 (20.6) | 0.204 |
| **Comorbidities, n (%)** |  |  |  |  |
| Anemia | 712 (69.3) | 348 (67.7) | 364 (70.8) | 0.279 |
| Hypertension | 253 (24.6) | 123 (23.9) | 130 (25.3) | 0.612 |
| AKI | 99 (9.6) | 51 (9.9) | 48 (9.3) | 0.751 |
| **Clinical outcome** |  |  |  |  |
| Hypernatremia | 43 (4.2) | 15 (2.9) | 28 (5.5) | 0.043 |
| Hypokalaemia | 92 (9.0) | 35 (6.8) | 57 (11.1) | 0.016 |
| Hypocalcemia | 120 (11.7) | 37 (7.2) | 83 (16.2) | <0.001 |
| Hospital LOS (day) | 12 (7-19) | 12 (7-19) | 12 (6-19) | 0.307 |
| 30 days mortality, n (%) | 32 (3.1) | 15 (2.9) | 17 (3.3) | 0.719 |
| Hospital mortality, n (%) | 33 (3.2) | 17 (3.3) | 16 (3.1) | 0.860 |

AKI, acute kidney injury; APTT, activated partial thromboplastin time; BC, bicarbonate concentration; LOS, length of stay; PICU, pediatric intensive care unit; SB, sodium bicarbonate; SICU, surgery intensive care unit; WBC, white blood cell.
